# Engineered phalangeal grafts for children with symbrachydactyly: A proof of concept

**DOI:** 10.1177/20417314241257352

**Published:** 2024-06-12

**Authors:** Romain Schaller, Adrien Moya, Gangyu Zhang, Mansoor Chaaban, Robert Paillaud, Ewelina M Bartoszek, Dirk J Schaefer, Ivan Martin, Alexandre Kaempfen, Arnaud Scherberich

**Affiliations:** 1 Department of Biomedicine, University Hospital Basel, University of Basel, Basel, Switzerland; 2 Department of Plastic, Reconstructive, Aesthetic and Hand Surgery, University Hospital Basel, Basel, Switzerland; 3 Paediatric Orthopaedic, University Children’s Hospital Basel, Basel, Switzerland

**Keywords:** Adipose-derived stromal cells, endochondral ossification, collagen sponges, preclinical model, symbrachydactyly and congenital bone defects

## Abstract

Tissue engineering approaches hold great promise in the field of regenerative medicine, especially in the context of pediatric applications, where ideal grafts need to restore the function of the targeted tissue and consider growth. In the present study, we aimed to develop a protocol to engineer autologous phalangeal grafts of relevant size for children suffering from symbrachydactyly. This condition results in hands with short fingers and missing bones. A previously-described, developmentally-inspired strategy based on endochondral ossification (ECO)—the main pathway leading to bone and bone marrow development—and adipose derived-stromal cells (ASCs) as the source of chondroprogenitor was used. First, we demonstrated that pediatric ASCs associated with collagen sponges can generate hypertrophic cartilage tissues (HCTs) *in vitro* that remodel into bone tissue *in vivo* via ECO. Second, we developed and optimized an *in vitro* protocol to generate HCTs in the shape of small phalangeal bones (108–390 mm^3^) using freshly isolated adult cells from the stromal vascular fraction (SVF) of adipose tissue, associated with two commercially available large collagen scaffolds (Zimmer Plug^®^ and Optimaix 3D^®^). We showed that after 12 weeks of *in vivo* implantation in an immunocompromised mouse model such upscaled grafts remodeled into bone organs (including bone marrow tissues) retaining the defined shape and size. Finally, we replicated similar outcome (albeit with a slight reduction in cartilage and bone formation) by using minimally expanded pediatric ASCs (3 × 10^6^ cells per grafts) in the same *in vitro* and *in vivo* settings, thereby validating the compatibility of our pediatric phalanx engineering strategy with a clinically relevant scenario. Taken together, these results represent a proof of concept of an autologous approach to generate osteogenic phalangeal grafts of pertinent clinical size, using ASCs in children born with symbrachydactyly, despite a limited amount of tissue available from pediatric patients.

## Introduction

Symbrachydactyly is a rare, congenital, and typically unilateral limb abnormality, presenting brachydactyly, cutaneous syndactyly and global hypoplasia of the hand which occurs in 1 in 20–30,000 live births.^1^ Patients suffer from a variety of phenotypes, ranging from having only short and sometimes webbed fingers (syndactyly) to no hand at all. In some cases, patients have an underdeveloped skeleton, but sufficiently long skin pockets to accommodate stable bone (for review).^1–4^ Traditionally, the hands of these children are improved with surgery to lengthen fingers or create a grip. However, each patient is treated according to its functional and aesthetic needs and resources of the healthcare system to create a grip or to improve hand function.^5^ Vascularized free toe transfers and free, non-vascularized phalangeal transfers as well as bone lengthening or skin deepening (web release) procedures have been used for this purpose. Each procedure has benefits and downsides. In particular, the autologous free non-vascularized phalangeal transfer is discussed intensively.^6–8^ On one hand, it helps to fill empty skin pockets and therefore lengthen the digit whereby it reasonably improves function in pinch and gross grip. On the other hand, it leads to an inacceptable donor site morbidity, especially if multiple toes are transferred, that worsens on the long term follow-up with the growth of the child.^6^

Developmental engineering uses a cell’s potential to recapitulate certain steps of a naturally occurring process involved in development and regeneration.^9^ Hence, we are proposing a developmentally inspired tissue engineering (TE) strategy that recapitulates endochondral ossification (ECO), the main pathway leading to bone and bone marrow (BM) development,^10,11^ to generate osteogenic grafts suitable for the treatment of 1- to 5-year-old children suffering from symbrachydactyly (i.e., 75–500 mm^3^—cylindrical grafts 4–8 mm diameter and 6–10 mm length). During ECO, mesenchymal stromal cells (MSCs) first condense, differentiate into chondrocytes and then progressively acquire a hypertrophic phenotype which is associated with the production of catabolic cytokines and angiogenic factors. As a result, the cartilage structure is invaded by vascular, osteogenic, and hematopoietic progenitors and is eventually remodeled into a bone organ with the presence of bone marrow elements.^12,13^

Several groups have successfully recapitulated ECO processes with various cell types including, (i) embryonic stem cells (ESCs),^14^ (ii) ESCs-derived MSCs,^15^ (iii) BM-MSCs,^16,17^ (iv) periosteum-derived cells (PDCs),^18,19^ (v) human adipose derived stromal cells (ASCs),^20–24^ (vi) chondrocytes^25,26^ or cell-free hypertrophic cartilage extracellular matrix (ECM).^27,28^ However, so far, the size of the resulting bone organs is often too small (i.e., a few mm^3^) for clinical translation. In 2015, Sheehy et al.^29^ used a BM-MSC-laden alginate hydrogel to generate hypertrophic cartilage tissues (HCTs) anatomically shaped in distal phalanges. In this study, the shape and volume of the graft were clinically relevant (around 350 mm^3^), but the resulting ECO was not optimal, with only the outer part of the implanted grafts able to remodel into bone tissue after subcutaneous implantation in immunodeficient mice.

Despite the great promise that TE holds for pediatric patients (e.g., the generation of grafts that would not only restore the function of the targeted tissue but also grow in concert with the child’s development^30^) reports in the literature using pediatric progenitor cells are scarce compared to the plethora of studies on adult stem/progenitor cells. One explanation for this lack of studies is the limited amount of tissue that can be used as cell source.^31^ Some reports in the literature suggest that pediatric BM-MSCs could be good candidates for TE applications because they possess a faster proliferating rate, significant morphology differences,^32^ lower senescence^33^ and retain a better osteogenic potential than adult ones.^34^ However, the BM-MSCs invasive harvesting procedure and limited abundancy^35^ are serious limitations for their use in pediatric TE approaches.

In a possible clinical scenario, syndactyly of the patients is released first and a full thickness skin graft from the groin region is then used to cover skin defects. At that time, the underlying adipose tissue (2–5 mL depending on the age of the patient) is resected from the skin graft without additional morbidity. Therefore, it represents an attractive source of mesenchymal stromal progenitors contained within the stromal vascular fraction (SVF) of the adipose tissue.^36,37^ The potential use of SVF cells and their expanded progeny (ASCs) for bone regeneration following an ECO pathway has been demonstrated by our group both in ectopic^20,21,23,24^ and orthotopic^38^ settings but so far only with material obtained from adult individuals.

In the present study, we thus wanted to generate the proof-of-concept of an autologous-based approach to generate osteogenic phalangeal grafts for the treatment of children suffering from symbrachydactyly following an ECO pathway. First, we investigated the ECO capabilities of pediatric ASC in a well-established model.^20,24^ Second, we developed a protocol using adult freshly isolated SVF cells to generate HCT grafts of clinical relevance (i.e., resembling the size and shape of a small phalangeal bone). Third, we validated our strategy using minimally expanded ASCs obtained from a 20-month-old donor.

## Materials and methods

### *In vitro* cell isolation and expansion

Adipose tissue (2–7.5mL) from four pediatric donors were obtained (15–29-month-old). Pediatric SVF cells were recovered following a protocol previously described.^39^After isolation, pediatric SVF cells were immediately seeded at a density of 10,000 cells/cm^2^ in complete medium (CM), consisting of α-MEM (Gibco, 22571-038) supplemented with 10% fetal bovine serum (FBS, Gibco, 10270-106), 1% HEPES (Gibco, 15630-056), 1% sodium pyruvate (Gibco, 11360-039), 1% penicillin–streptomycin–glutamine (Gibco, 10378-016), as well as 5 ng/ml FGF2 (Bio-Techne, 233-FB-025) and further cultured at 37°C, 5% CO_2_ and 95% humidity with media change twice a week. Upon reaching confluency (90%), pediatric cells were detached with 0.05% trypsin/0.01% EDTA (Gibco, 25300-054) and seeded again at 3000 cells/cm^2^ for one additional cell passage. The resulting population was termed pediatric ASC P1. Adult SVF cells—obtained from 2 donors (37- and 77-years old)—used in the present study were isolated following the same protocol but not expanded *in vitro*.

### *In vitro* generation of HCTs

Small HCTs were generated from pediatric ASC P1 (0.5 × 10^6^ cells) associated with cylindrical (3 mm and a diameter of 4 mm, total volume 37.7 mm^3^) collagen sponge (Avitene™ Ultrafoam™ Collagen Sponge from Becton Dickinson, 1050050) as previously described^24^ and serially exposed to chondrogenic factors for 3 weeks followed by hypertrophic factors for 1 week. For Zimmer phalangeal HCTs fabrication, 3 × 10^6^ (2000 cells/mm^3^) or 9 × 10^6^ (6000 cells/mm^3^) of either SVF adult cells or pediatric ASC P1 were seeded onto Zimmer plug^®^ on a non-crosslinked collagen type I of porcine origin (Zimmer, 0102Z collagen plug) shaped in a cylindrical plug of 2 cm height and 1 cm diameter (total volume 1570 mm^3^). For Optimaix phalangeal HCTs fabrication, 0.3 × 10^6^ (2000 cells/mm^3^) or 0.9 × 10^6^ (6000 cells/mm^3^) of SVF adult cells were seeded onto a crosslinked collagen type I matrix of porcine origin with highly oriented fibers (Optimaix 3D^®^, Matricel GmbH), cut in rectangular bars 10 × 5 × 3 mm (volume 150 mm^3^). Pediatric ASC P1 were not used with the Optimaix 3D scaffolding material. Next, cell-laden sponges were serially exposed to chondrogenic factors for 3-,4- or 6-weeks followed by hypertrophic factors for 1 week. Briefly, the chondrogenic induction medium was composed of serum-free culture medium composed of Gibco Dulbecco’s Modified Eagle Medium (DMEM, Gibco, 10938-025), 1% HEPES (Gibco, 15630-056), 1% sodium pyruvate (Gibco, 11360-039), 1% penicillin–streptomycin–glutamine (Gibco, 10378-016), ITS+1 (Sigma, I2521), and 1.25 mg/ml human serum albumin (CSL Behring, 43075). The serum-free culture medium was further supplemented with 10^−7^ M dexamethasone (Sigma, D-2915), 0.1 mM ascorbic acid (Sigma, A-8960), 10 ng/ml transforming growth factor-β3 (TGF-β3, PeproTech, 100-36E), and 10 ng/ml bone morphogenetic protein 6 (BMP-6, PeproTech, 120-06). The hypertrophic induction medium was composed of serum-free culture medium supplemented with 0.1 mM ascorbic acid (Sigma, A-8960), 10 mM β-glycerophosphate (Sigma, G9422), 10^−8^ M dexamethasone, 50 mM L-thyroxin (Sigma, T-1775), and 50 pg/ml interleukin1-β (Sigma, SRP3083).

### *In vitro* volumetric quantification

Volumetric quantification of the *in vitro* generated phalangeal HCTs was performed using the open-source software FIJI version 2.1.0. on macroscopic cell culture images. Briefly, for the Optimaix-based HCTs, the length, width, and height (mm) were measured and the volume (mm^3^) was calculated using the formula:

V(OptimaixHCTs)=length×width×height

For the Zimmer-based HCTs, the length and diameter were measured (mm) and the volume (mm^3^) was calculated using the formula:

$$V\left(Zimmer\;HCTs\right)=length\;\times \pi \times {\left(\frac{diameter}{2}\right)}^{2}$$

The Zimmer-based HCTs that were bend out of cylindrical shape were removed from the volumetric analyses.

### *In vivo* ectopic implantation in nude mice

At the end of the cartilage maturation *in vitro*, HCTs grafts (maximum 4 per mouse) were implanted subcutaneously in athymic CD1 nu/nu female nude mice (Charles River Laboratories, Wilmington, MA). Mice were operated under the permission of the Federal Veterinary Office (permit BS 1797) as previously described.^20^ In total, 12 mice were implanted with 4 pediatric donors and 2 adult donors as described in Table 1.

**Table 1.** Summary of implanted HCTs.

Cell type	Donor#	Scaffold-HCT	Mice	Replicates	Figure
Pediatric ASC P1	1	Ultrafoam 4 mm	1	*N* *=* 3	Figure 1
Pediatric ASC P1	2	Ultrafoam 4 mm	1	*N* *=* 4	Figure 1
		Zimmer-7 W	2	*N* *=* 8	Figure 6; Supp. 3
Pediatric ASC P1	3	Ultrafoam 4 mm	1	*N* *=* 2	Figure 1
Pediatric ASC P1	4	Ultrafoam 4 mm	1	*N* *=* 4	Figure 1
Adult SVF cells	1	Zimmer-5 W	1	*N* *=* 4	Figure 4(C–E); Supp. 2
		Zimmer-7 W	1	*N* *=* 4	Figure 4
		Optimaix-5 W	1	*N* *=* 4	Figure 4(C–E); Supp. 2
		Optimaix-7 W	1	*N* *=* 4	Figure 4
Adult SVF cells	2	Zimmer-7 W	1	*N* *=* 4	Figure 4
		Optimaix-7 W	1	*N* *=* 4	Figure 4

### Microcomputed tomography

After explantation, samples were retrieved and fixed overnight in 4% formalin (Formafix). Then, microcomputed tomography (microCT) data were acquired by using a high-resolution scanner (SkyScan1172, Skyscan, Belgium) and 0.5-mm aluminum filtered X- rays (applied voltage 50 kV; current, 200 uA). Transmission images were acquired during a 360° scan rotation with an incremental rotation step size of 0.25°. Reconstruction was performed using a modified Feldkamp algorithm at an isotropic voxel size of 4 μm for the ultrafoam^®^-based HCTs and 10 μm for the Zimmer- and Optimaix-based HCTs. Three-dimensional rendering, thresholding, segmentation, and 3D measurements were performed using VG Studio MAX 2.2 software (Volume Graphics, Heidelberg, Germany).

### Histological assessments

For histological analyses, fixed samples were decalcified by 10% EDTA solution (MoL-Decalcifier, Milestone srl) if necessary, and embedded in paraffin. The scaffolds were cut into 5 µm thick sections using a Microtome HM 355S (Thermo Scientific) and the sections were placed onto Poly-lysine slides (Thermo Scientific, Waltham, US). The tissue sections were deparaffinized and rehydrated then stained with Haematoxylin-Eosin (both from Sigma Aldrich) and Safranin-O (Sigma-Aldrich, 84120) using the Epredia™ Gemini™ AS Automated Slide Stainer. Collagen type II (Reactivity: human, MP Biomedicals, 63171) and collagen type X (Reactivity: human; Cat. no. 14-9771-80, Invitrogen), and Bone sialoprotein (BSP, Reactivity: human; Cat. no. 52128, Abcam) stainings were performed with Ventana Discovery Ultra (RocheDiagnostics (Switzerland) SA) automated slide stainer. In brief, tissue sections were deparaffinized and rehydrated. Antigens were retrieved by a protease (Protease 3, ref. 760-2020, Ventana) digestion for 20–44 min at 37 °C. Primary antibody was manually applied and incubated for 1 h at 37°C. After washing, the secondary antibody was incubated for 1 h at 37°C. Detection step was performed with the Ventana DISCOVERY ChromoMap DAB (ref. 760-159 Ventana) detection kit. Afterward, the slides were counterstained with hematoxylin II, followed by the bluing reagent (respectively, Cat. no. 790-2208 and 760-2037, Ventana). Sections were then dehydrated, cleared, and mounted with permanent mounting and coverslips. Images of the histological sections were acquired with a Nikon Ti2 widefield microscope, a Nikon DS-Ri2 camera and a CFI Plan Apo Lambda NA 0.75, 20× objective. The software used was the NIS-Elements AR 5.21.03.

### Human nuclei immunofluorescence staining

Decalcified samples were cryoprotected with sequential incubations in 15% and 30% sucrose/PBS solutions at 4℃ overnight. Samples were embedded in OCT compound, cryosectioned into 10 µm sections, and stored at −80℃ until use. Sections were rehydrated with PBS for 10 min at room temperature, permeabilized with 0.3% Triton X-100/PBS, and then subjected to 1% BSA and 5% goat serum to block unspecific binding. Then, sections were incubated with a 1:100 dilution of anti-human nuclei antibody (Millipore, MAB1281) overnight at 4°C and further incubated with a 1:500 dilution of Alexa Fluor 546 goat anti-mouse IgG (H + L) secondary antibody (Invitrogen A11030) and 1:1000 DAPI (4′,6-diamidino-2-phenylindole, Invitrogen D1306) to stain nuclei prior to imaging.

### Automated *in vitro* cartilage maturation score

To grade the maturity of *in vitro* engineered cartilage, the Automated Modified Bern Score was used to score histological images.^40^ A python script to grade whole slide images was used along with the trained model for visualization of the scores within a construct (https://github.com/Lopo358/AMBS_whole_slide_image).

### QuPath analyses

Tissue classification into four classes, namely: bone, bone marrow, cartilage and fibrotic tissue, was performed as previously described^24^ using the open-source software QuPath v0.3.2.^41^ In short, a training image was composed of multiple regions of interest from representative scans to account for tissue variability, and annotations for each class were manually drawn. First, tissue was classified into two regions: Cartilage/Bone area and Bone marrow/Fibrotic tissue area. Each of these regions were further classified into single region annotations, using parameters: Pixel_classifier_type: “OpenCVPixelClassifier,” Resolution: moderate: (2.93 μm/px), Channels selected (Red, Green, Blue); Scale: 1.0. Features used: “gaussian,” “weighted_std_dev,” “gradient magnitude,” “Laplacian,” “structure_tensor_eigenvalue_max. Results were inspected by an expert and manually corrected if needed. Corresponding training images and classifiers can be found on Zenodo (https://zenodo.org/records/10680128).

## Results

### Pediatric ASCs are suited for bone tissue engineering via the ECO pathway

**Figure 1.**
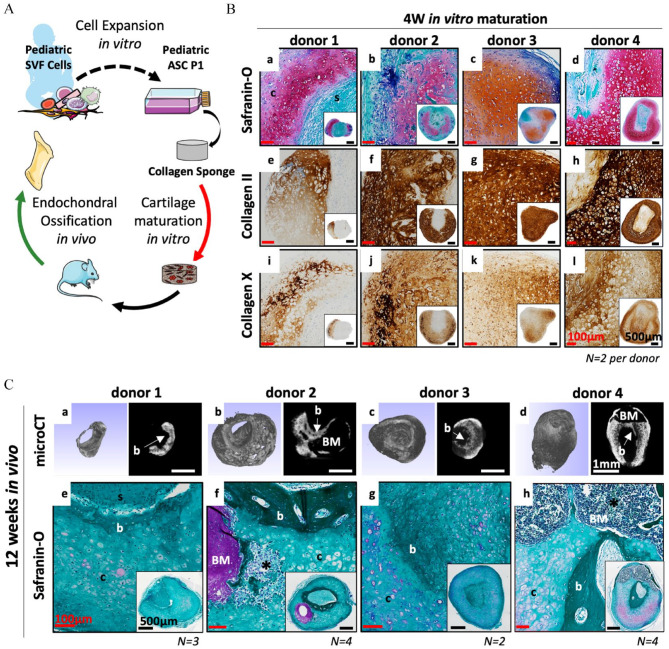
Pediatric ASCs recapitulate endochondral ossification *in vivo* when adequately primed *in vitro.* (A) Schematic overview of the experimental design. (B) 0.5 × 10^6^ Pediatric ASC P1 cells are associated with a collagen scaffold (cylindrical shape, 4 mm diameter and 3 mm height; *V* = 37.68 mm^3^) and cultured *in vitro* for 4W (3 weeks chondrogenic + 1 week hypertrophy). Representative images of (a–d) Safranin-O staining, cartilage tissue in red, mineralized cartilage in blue and non-cartilage tissue in green, (e–h) collagen type II and (i–l) collagen type X of the *in vitro* generated cartilage tissues. (C) *In vivo* bone remodeling via endochondral ossification after 12 weeks of ectopic implantation in a nude mice model. Representative images of (a–d) Microcomputed tomography (µCT), Bone (dense signal with smooth surface) mineralized HCT (signal with a rough surface). (e–h) Safranin-O staining, bone (dark green), fibrotic tissue (light green), cartilage (red), bone marrow (purple). (*N* *=* *13, 2–4 biological replicates per donor, 4 donors tested).* Black scale bar = 500 µm, white scale bar = 1 mm, red scale bar 100 µm. Symbols: b: Bone, BM: Bone Marrow, c: cartilage, s: scaffold, *: blood vessel.

We first addressed whether ASCs obtained from pediatric adipose samples (12–29-month-old) could recapitulate ECO in a similar fashion to what has been demonstrated for adult ASCs.^20,24^ To that aim, Pediatric ASCs were associated with collagen sponges (UltraFoam^®^ with a cylindrical shape; *h* = 3 mm *d* = 4 mm *V* = 37.7 mm^3^) and cultured for 4W (serially exposed to chondrogenic factors for 3 weeks followed by exposure to hypertrophic factors for 1 week). The bone forming capacity of these *in vitro* generated HCTs was assessed *in vivo* in an ectopic immunodeficient mouse model for 12 weeks. Figure 1(A) offers a summary of the experimental set-up. Cells from each tested donor (*N* = 4) were capable of forming cartilage tissues *in vitro* as evidenced by round chondrocytes within lacunae embedded in ECM positive for glycosaminoglycans (GAG, in red), collagen II and collagen X, a marker of hypertrophy (Figure 1(B)). The periphery of each HCTs displayed mineralization (in blue) while their core was mostly comprised of non-chondrogenic cells and collagen sponge remanence (Figure 1(Ba–d)).

After implantation, both the micro-computed tomography (µCT) and the histological analyses demonstrated successful and reproducible bone formation *in vivo*. For every donor tested, newly formed bone tissue was located at the core of the implanted constructs and characterized by a smooth surface on the µCT images (Figure 1(Ca-d)) and by a darker green color on the safranin-O images. The persistence of cartilage tissues was observed and characterized by chondrocytes embedded in a faintly GAG positive ECM (in red) (Figure 1(Ce–h)). In contrast, the ECO was most advanced for donor-2 and -4, where a larger amount of bone as well as the presence of bone marrow compartment within the bone ossicles formed were observed (Figure 1(Cb,f and 1Cd,h)). These results are in line with our previous report using a similar experimental set-up with adult cells^24^ and demonstrate that ASCs derived from pediatric adipose tissues can similarly generate hypertrophic cartilage *in vitro* that remodel into bone tissue via ECO *in vivo*.

### Adult SVF cells in larger collagen sponges generate phalangeal HCT grafts suited for the treatment of children suffering from symbrachydactyly

**Figure 2.**
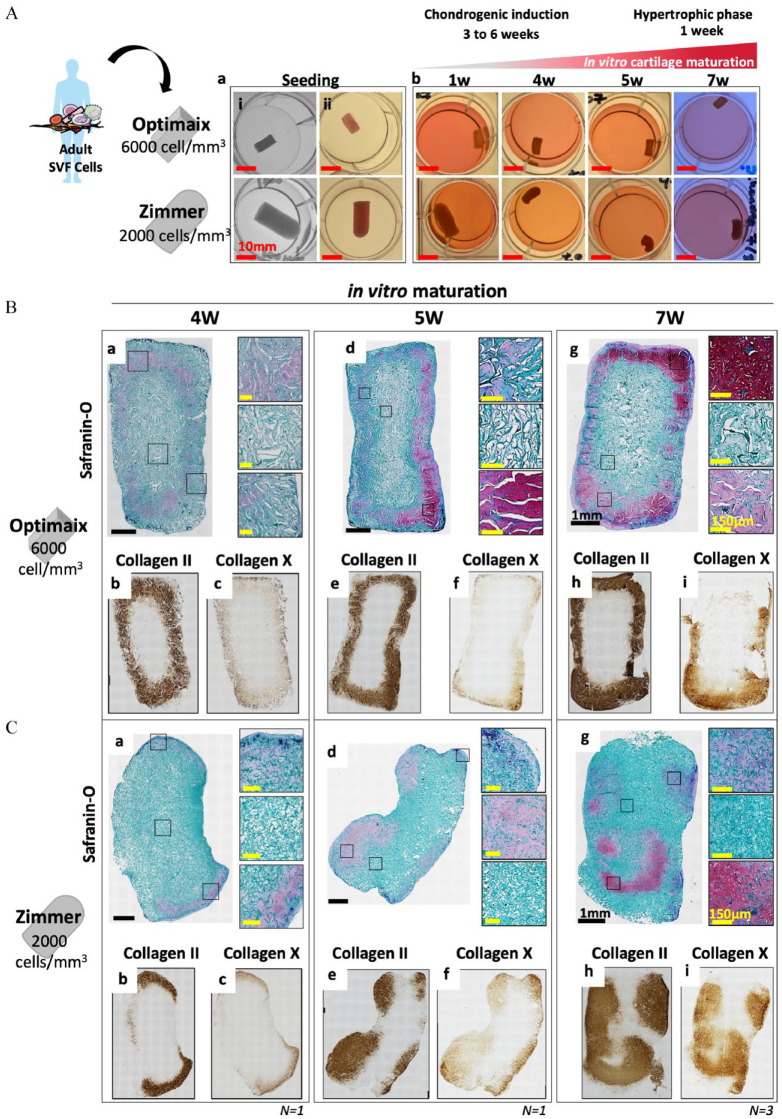
Adult SVF cells associated large collagen sponge scaffolds generate phalangeal HCT grafts *in vitro*. (A) Schematic overview of the experimental design and macroscopic views of the phalangeal HCT grafts (a) (i) before and (ii) after cell seeding and (b) throughout the *in vitro* cartilage maturation. Histological assessments of (B) 0.9 × 10^6^ SVF cells (6000 cell/mm^3^) associated with Optimaix^®^ collagen scaffold (bar shape, 10 mm length 5 mm width and 3 mm height; *V* = 150 mm^3^) or (C) 3 × 10^6^ SVF cells (2000 cell/mm^3^) associated with Zimmer Plug^®^ collagen scaffold (cylindrical shape, 10 mm diameter and 20 mm length; *V* = 1500 mm^3^) and cultured *in vitro* for (a–c) 4W (3 weeks chondrogenic + 1 week hypertrophy), (d–f) 5 W (4 weeks chondrogenic + 1 week hypertrophy) or (g–i) 7W (6 weeks chondrogenic + 1 week hypertrophy). Representative images of (a,d,g) Safranin-O staining, (b,e,h) collagen type II and (c,f,h) collagen type X obtained from the *in vitro* generated HCT grafts. *(N* *⩾* *1, 1–2 biological replicates per donor, 1–2 donors tested).* Red scale bar = 10 mm, black scale bar = 1 mm, yellow scale bar = 150 µm.

To generate phalangeal HCT grafts, we tested the influence of (i) the initial size and composition of the scaffolding material, (ii) the cell seeding density and (iii) the *in vitro* culture duration. Specifically, we tested the commercially available Zimmer Plug^®^ (a non-crosslinked collagen type I of porcine origin similar to the UltraFoam^®^ shaped in a plug—*h* = 20 mm *d* = 10 mm *V* = 1500 mm^3^) and Optimaix 3D^®^ (a crosslinked collagen type 1 matrix of porcine origin with highly oriented fibers shaped in form of a bar—*l* = 10 mm *w* = 5 mm *h* = 3 mm *V* = 150 mm^3^) associated with either 2000 or 6000 SVF cells/mm^3^ and cultured for 4W, 5W, or 7W *in vitro* (chondrogenic factors for 3–6 weeks followed by hypertrophic factors for 1 week). For the two adult donors tested, both scaffolding materials were capable of supporting a gradual cartilage formation *in vitro* (Figure 2). Interestingly, with the higher cell density, the strongest cartilage formation (i.e., more area positive for GAG, collagen II, and collagen X) occurred in the Optimaix-based grafts, whereas cartilage formation was impaired in Zimmer-based grafts (Figure S1). As expected from his crosslinked nature, the Optimaix-based grafts were able to retain their shape and size throughout the *in vitro* maturation (Figure 2(A)). In addition, histological analyses revealed that the cartilage formation was located exclusively at the periphery while the core of these grafts was comprised of non-chondrogenic cells attached to the scaffolding material (Figure 2(B)).

In contrast, the Zimmer-based grafts had a behavior more similar to the ones based on smaller UltraFoam-based constructs used in previous studies,^20,24^ contracting rapidly during the first 4 weeks of *in vitro* maturation, with initial formation of cartilage at the periphery of the grafts (Figure 2(Ca–c)). At later time-points, the cartilage pockets were larger and also present in the inner parts of the constructs. However, even after 7 weeks of *in vitro* maturation, non-cartilaginous areas remained (Figure 2(Cg–i)).

**Figure 3.**
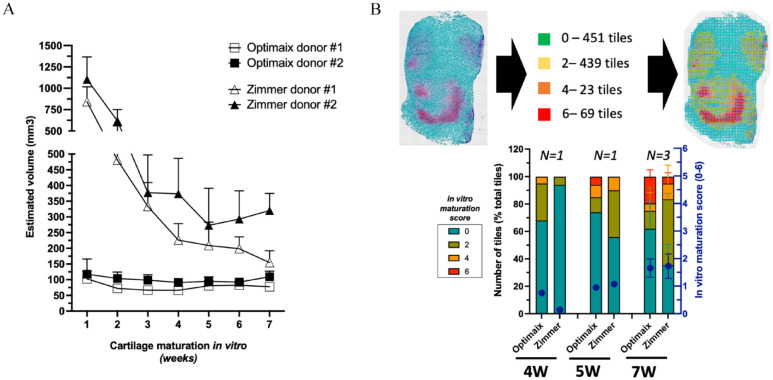
*In vitro* evolution of the size and cartilage maturation of phalangeal HCTs grafts generated from adult SVF cells. (A) Time course of the estimated volumes of the phalangeal HCT grafts for both the Zimmer Plug® and Optimaix^®^ groups. Data are expressed as mean ± SD (mm^3^). *(N* *=* *6, 6 biological replicates per donor, 2 donors tested per scaffolding material).* (B) Automated histological assessment of the *in vitro* cartilage maturation for the 4W-, 5W- and 7W-groups. Safranin-O-stained pictures are cut into square tiles (336 pixels by 336 pixels) and scored 0-non cartilaginous tissue; (2) poor cartilage maturation; (4) moderate cartilage maturation; (6) high cartilage maturation. Distribution of the number of tiles for each cartilage maturation category expressed as a percentage of the total number of tiles and plotted as a histogram. *(N* *⩾* *1, 1–2 biological replicates per donor, 1–2 donors tested).*

When quantifying the contraction of each graft throughout the *in vitro* phase, a 70% shrinkage of Zimmer-based grafts was observed during the first weeks of culture followed by a plateau concomitant with the appearance of cartilage tissues which occurred around week-4 or -5, depending on the donor (Figure 3A). The Optimaix-based grafts, due to their highly oriented fibers, showed a much more limited contraction (10%) during the *in vitro* phase. After 7 weeks of maturation, the final volume ranged from 59 to 136 mm^3^ for Optimaix-based grafts, and 108 to 390 mm^3^ for the Zimmer-based ones (Figure 3A). To quantify the cartilage maturation in the phalangeal grafts, we used a modified version of an automated deep-learning method previously developed in our laboratory.^40^ In a previous report, this maturation index was shown to be predictive of the bone remodeling via ECO, with an optimal result for scores ranging from 1 to 3 (out of a maximal score of 6).^24^ While the overall maturation index was similar for both materials, with a gradual score increase reaching 1.7 after 7 weeks, their cartilaginous maturation profiles were different. The Optimaix-based grafts displayed a more heterogenous profile with a marked difference between non cartilaginous areas (62 ± 19.8% 0-scored tiles) and strongly differentiated regions (19.5 ± 4.9% 6-scored tiles). Whereas, the Zimmer-based grafts were more homogeneous and contained fewer non cartilaginous areas (35.5 ± 14.8% of 0-scored tiles) (Figure 3B).

**Figure 4.**
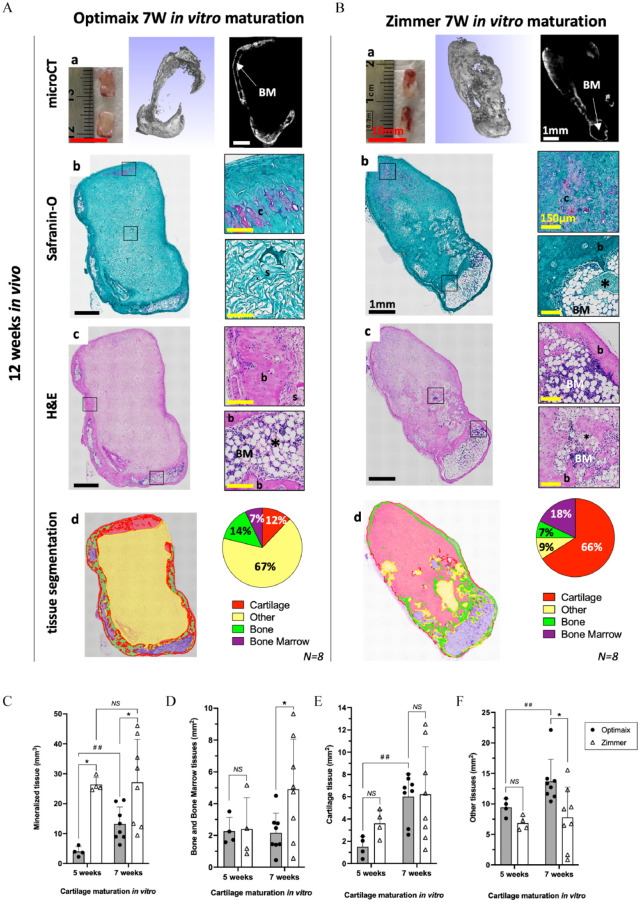
Phalangeal HCT grafts remodel into bone organs via endochondral ossification *in vivo*. Bone remodeling phenotypes observed for (A) 7W-Optimaix- and (B) 7W-Zimmer-based grafts after 12 weeks of implantation *in vivo*. (a) Macroscopic and Micro-µCT views. Representative images of (b) Safranin-O and (c) H&E sections. (d) Automated tissue segmentation performed H&E sections using a semi-automated script on QuPath. (C) Evolution of mineralized tissue (mm^3^) obtained by µCT after 12 weeks *in vivo*. Data are expressed as mean ± SD *(N ⩾ 4, 4 biological replicates per donor, 1-2 donors tested)*. Evolution of (D) Bone and Bone Marrow, (E) Cartilage and (F) Other tissues within the H&E sections obtained after 12 weeks *in vivo*. Data are expressed as a percentage of the total tissue mean ± SD. *(N* *⩾* *4 biological replicates per donor, 1–2 donors tested).* b: Bone, BM: Bone Marrow, c: cartilage, s: scaffold, *: blood vessel. Red scale bar = 10 mm, black scale bar = mm, yellow scale bar = 150 µm. *p < 0.05 *Mann Whitney test (Zimmer group vs. Optimaix group);* ^##^*p* *<* *0.01 Mann Whitney test (7 weeks vs. 5 weeks).*

Next, we tested the bone forming capacity of these phalangeal HCT grafts in an ectopic, immune-compromised mice model mimicking the clinical scenario of an empty phalangeal pocket. Briefly, Zimmer- and Optimaix-based grafts matured for either 5W or 7W were implanted subcutaneously for 12 weeks in nude mice. Samples were then collected and the bone formation was analyzed by µCT and histological analyses. All constructs tested were successful in achieving bone formation via ECO. The *in vivo* results obtained for 7W-Optimaix- and -Zimmer-based grafts are presented in Figure 4, whereas the ones of 5W-Optimaix- and -Zimmer-based grafts are presented in Supplementary Figure 2. At time of explantation, we could macroscopically observe vascularization of the implanted cartilage constructs both for the 7W-Optimaix and -Zimmer groups (Figure 4(A)). In addition, µCT analyses showed strong mineralization and the presence of bone marrow pockets for both groups (Figure 4(Aa and Ba)). Endochondral ossification of the implanted cartilage was highlighted by the presence of interlaced bone and cartilage tissue (Figure 4(Ab–c and Bb–c)). For the 7W-Optimaix group, the bone formation was located solely at the periphery of the construct (where cartilage was initially present at the time of implantation) and contained bone marrow pockets (in 2 out of 8 constructs) while the vast majority (67%) of the construct was still comprised of non-remodeled Optimaix sponge (Figure 4(Ab–d)). In contrast, the 7W-Zimmer group showed better remodeling *in vivo*, with most of the construct comprised of either bone, bone marrow (in 3 out of 8 constructs) or cartilage tissues (Figure 4(Bb–d)). In both cases, the cartilage remanence was characterized by a faintly positive safranin-O staining still present after the 12 weeks of implantation (Figure 4(Ab and Bb)). Similar results (i.e., presence of bone and cartilage tissues after 12 weeks of implantation) were obtained for the 5W-groups, however we could not detect bone marrow tissues in any of them (Figure S2).

Volumetric quantification of the mineralized tissues by µCT demonstrated a superior osteogenic potential of the Zimmer-based grafts as compared to the Optimaix ones, specifically, with 26.4 ± 2.4 vs 4.0 ± 1.6 mm^3^ and 27.2 ± 14.3 vs 13.3 ± 5.6 mm^3^ of mineralized tissues obtained for the 5W- and 7W-*in vitro* cartilage maturation, respectively (*p* < 0.05; Figure 4(C)). On the H&E sections analyzed for tissue segmentation, we found no difference in the amount of bone and bone marrow present in the 5W-grafts, however, there was significantly more bone and bone marrow tissues in the 7W-Zimmer group (4.9 ± 3.2 mm^3^) compared to the 7W-Optimaix group (2.1 ± 1.2 mm^3^; Figure 4(D)). Conversely, for these two groups we could observe a significant increase in other tissues (i.e., non-cartilaginous and non-bony) for the 7W-Optimaix group (13.7 ± 3.6 mm^3^) compared to the 7W-Zimmer group (7.8 ± 5.0 mm^3^; Figure 4(F)). Finally, while we observed an increase in cartilage content in the 7W-constructs when compared to the 5W-constructs, no significant differences were observed between the Optimaix and Zimmer groups (Figure 4(E)). We thus were able to generate grafts shaped in the form of a phalanx from adult SVF cells with both scaffolding materials. However, after 12 weeks of implantation, an optimal bone formation via ECO characterized by (i) the maintenance of shape and size of the implanted cartilage, (ii) the presence of bone tissue and bone marrow, and (iii) the remodeling of the collagen scaffold, was obtained for the 7W-Zimmer group.

### Pediatric ASCs in Zimmer plug sponges generate phalangeal HCT grafts *in vitro* that remodel into bone tissue via ECO *in vivo*

**Figure 5.**
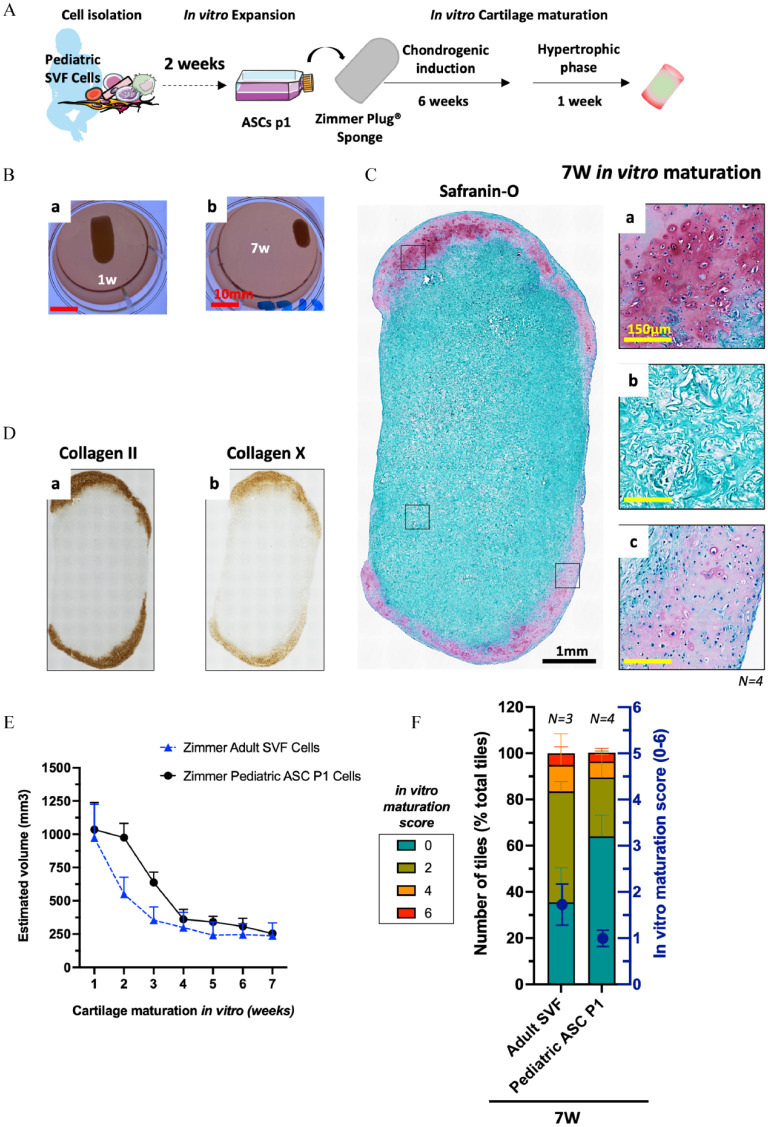
Envisioned manufacturing strategy for autologous phalangeal HCT grafts production to treat children suffering from Symbrachydactyly. (A) Schematic overview of the manufacturing protocol. Pediatric SVF cells are expanded for 2 weeks (ASC P1). 3 × 10^6^ pediatric ASCs P1 are associated with Zimmer Plug^®^ collagen scaffolds and cartilage maturation is induced for 7 weeks. (B) Macroscopic view of the cartilage constructs after (a) 1 week and (b) 7 weeks of cartilage maturation. (C) Representative images of Safranin-O staining, (D) (a) collagen type II and (b) collagen type X obtained from the *in vitro* generated cartilage. (E) Time course of the estimated volumes of the HCTs. Data are expressed as mean ± SD (mm^3^). *(N* *⩾* *4, 4–6 biological replicates per donor, 1-2 donors tested per cell group).* (F) Automated histological assessment of the *in vitro* maturation of the cartilage tissues after 7 weeks of induction. *(N* *⩾* *3, 1–3 biological replicates per donor, 1–2 donors tested).* Red scale bar = 10 mm, black scale bar = 1 mm, yellow scale bar = 150 µm.

Next, we wanted to demonstrate the feasibility of an autologous approach for the construction of phalanx in children suffering from Symbrachydactyly. We tested this clinical approach with a pediatric donor, 2.5 mL of adipose tissue were recovered from a 15-month-old donor and 1.95 × 10^6^ SVF cells were isolated by enzymatic digestion and cultured as monolayer for 2 weeks (3250 cells/cm^2^). After 2 weeks of expansion, more than 200 × 10^6^ cells (referred to as ASCs P1) were generated and the Zimmer plug collagen sponges were seeded with 3 × 10^6^ ASCs P1 (2000 cells/mm^3^). Following the same protocol as the one used for adult SVF cells, Zimmer-based cartilage grafts were generated *in vitro* and their bone forming capacity was evaluated *in vivo* in an ectopic immunodeficient mouse model. Figure 5(A) offers a summary of the *in vitro* manufacturing protocol. After 7 weeks of *in vitro* cartilage maturation, a contraction of the Zimmer collagen sponge was observed (Figure 5(B)) as well as the presence of cartilage tissue, which formed a shell (pink and red areas on the safranin-O staining) located at the periphery of the Zimmer constructs, while the inner part of the constructs was mostly composed of compacted collagen sponge and few cells (Figure 5(Ca–c)). Likewise, the presence of collagen II and collagen X was limited to the periphery of the constructs (Figure 5(D)). The contraction of the construct throughout the *in vitro* cartilage differentiation was similar to the one observed for the adult-SVF cells (Figure 5(E)). Finally, using the automated *in vitro* maturation score, we could show a reduced chondrogenic capacity for the pediatric ASCs P1 (maturation score of 1 ± 0.2) when compared to the adult SVF (maturation score of 1.7 ± 0.4; Figure 5(F)).

**Figure 6.**
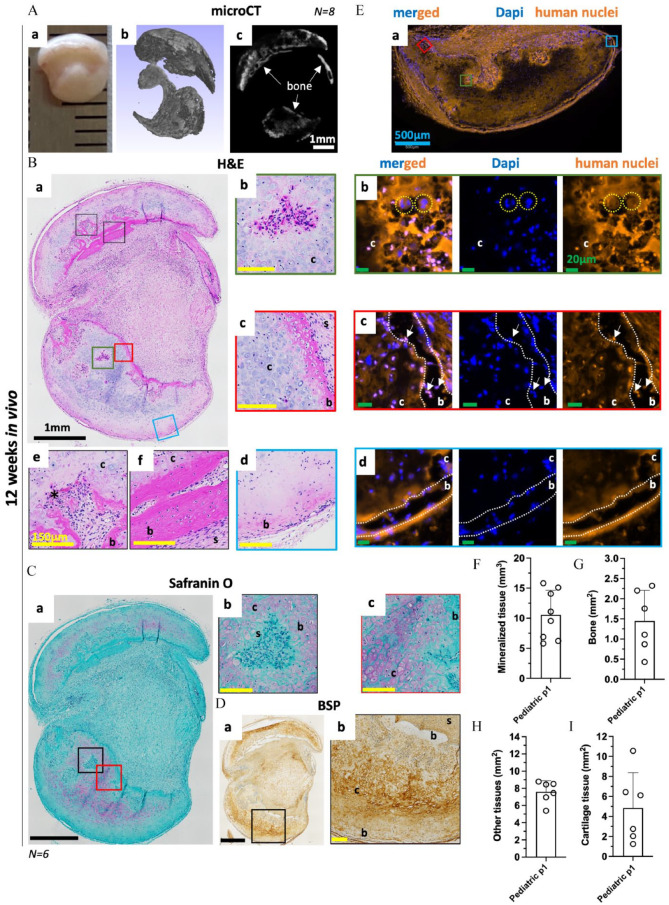
Pediatric phalangeal HCT grafts remodel into bone tissue via endochondral ossification. 7W Pediatric ASCs P1 based Zimmer constructs were implanted in an ectopic nude mice model for 12 weeks. (A) (a) Macroscopic and (b,c) Micro- µCT views post-implantation. Representative images of (B) H&E and (C) Safranin-O staining showing evidence of ECO in the implanted constructs (a) whole section, (b) early cartilage remodeling with the presence of multinucleated cells, (c) Chondrocyte to osteoblast transition, (d) outer cortical bone, (e) intermediate cartilage remodeling with the presence of bone and blood vessels and (f) mature bone located at the core of the constructs. (D) Bone Sialoprotein (BSP) staining (a) whole section, (b) early cartilage remodeling. (E) Mouse and human cells contribution to the ECO. Representative images of human nuclei and Dapi staining of (a) large section, (b) early cartilage remodeling with the presence of murine multinucleated cells, (c) chondrocyte to osteoblast transition with the presence of human chondrocytes and osteoblasts and (d) outer cortical bone with the presence of murine osteoblasts. Mouse nuclei in blue, and human nuclei in pink on the merged pictures. (F) Evolution of mineralized tissue in mm^3^ for pediatric phalangeal HCT grafts obtained by µCT after 12 weeks *in vivo*. Data are expressed as mean ± SD *(N* *=* *8, 1 donor tested)*. Evolution of (G) bone and bone marrow, (H) other and (I) cartilage obtained by automated tissue segmentation performed H&E sections using a semi-automated script on QuPath. Data are expressed as a percentage of the total tissue mean ± SD. *(N* *=* *6, 1 donor tested).* Symbols: b: bone, c: cartilage, s: scaffold, *: blood vessel, yellow circles murine multinucleated cells, white arrows human osteoblasts. White scale bar = 1 mm, black scale bar = 1mm, blue scale bar = 500 µm, yellow scale bar = 150 µm, green scale bar = 20 µm.

After 12 weeks of implantation, similarly to what was observed for the adult SVF cells-based Zimmer cartilage constructs, bone formation via ECO was obtained for the pediatric Zimmer-grafts (Figure 6). In some cases, we could macroscopically observe a reduction in the size of the implanted constructs (Figure 6(Aa)). For all constructs tested (*N* = 8), the presence of newly formed bone (characterized by a smooth surface with hollow sinuses) adjacent to the mineralized cartilage tissue was visible on the µCT images (Figure 6(Abc)). On H&E- and Safranin-O-stained sections of the explanted pediatric cartilage constructs, we observed not only the presence of vascularized bone tissue but also the presence of cartilage tissue containing numerous chondrocytes, some likely transitioning towards an osteoblastic lineage (Figure 6(B–D)). In addition, even after 12 weeks of implantation, the cartilage tissues exhibited a relatively high GAG content in the ECM and the presence of hypertrophic chondrocytes evidenced by the Safranin-O and Bone sialoprotein (BSP) staining, respectively (Figure 6(C,D) and Supplementary Figure 3). To investigate the contributions of the implanted cells (human) and the host cells (mouse) on the *in vivo* bone- and cartilage-formation, a human-nuclei staining on was performed on cryosections of pediatric Zimmer-based constructs (Figure 6(E)). The multi-nucleated osteoclastic cells present in small areas containing blood vessels inside the cartilage matrix were of mouse origin (Figure 6(Eb)), thus evidencing the host-mediated bone remodeling that is taking place (Figure 6(Bb and Be)). In the inner part of the constructs, mostly filled with human cells, a distinctive interphase between newly formed bone and the calcified cartilage matrices was observed with chondrocytes gradually replaced by osteoblasts (Figure 6(Bc and 6Ec)). In addition, a progressive loss of BSP expression was observed in the human implanted chondrocytes from the calcified cartilage towards the newly formed bone (Figure 6(Cb) and Supplementary Figure 3(c and d)). In contrast, for the newly formed cortical bone tissue obtain at the periphery of the constructs, the cells embedded in the bone matrix were of mouse origin, demonstrating that they were the one responsible for the endochondral ossification located on the outer part of the implanted constructs (Figure 6(Bd and Ed)).

In conclusion, we were able to demonstrate the feasibility of a clinical strategy using pediatric adipose derived cells to generate tissue engineered phalanx grafts for children suffering from symbrachydactyly.

## Discussion

In this study, we showed for the first time the ECO potential of pediatric ASC associated with small collagen sponge scaffolds and of phalangeal HCT grafts. These were generated from either adult SVF cells or pediatric ASCs associated with large collagen sponges in an ectopic, preclinical immunocompromised mouse model aiming at modeling both an autologous approach—with limited immune response—and the empty phalangeal pocket of children suffering from symbrachydactyly. We observed a small reduction in chondrogenicity for expanded pediatric ASCs as compared to adult SVF cells. However, they both remained capable of generating HCT grafts that successfully remodeled into bone organs, demonstrating the feasibility of an autologous approach to generate phalangeal substitutes.

Remarkably, after implantation, HCTs derived from pediatric donors not only displayed signs of ECO but also maintenance of cartilage tissues characterized by the presence of proliferating chondrocytes and a high content in GAG within the ECM (Figures 1 and 6). The latter features were absent from all the adult SVF-cells implanted HCTs tested. Similarly to what was previously observed for HCT based on BMSCs,^42^ the bone located at the core the implant and in direct contact with the cartilage tissue was of human origin (Figure 6), suggesting that some implanted hypertrophic chondrocytes likely could give rise to osteoblasts. Further studies are needed to confirm and understand the underlying mechanisms of this *in vivo* cartilage homeostasis and hypertrophic chondrocytes to osteoblasts transition observed in pediatric ASCs-derived HCTs.

The manufacturing protocol developed here was adopted to ensure a timely and feasible clinical translation. In particular, it involves (i) limited numbers of cells (3 × 10^6^ per graft), (ii) a simple, straightforward *in vitro* culture system, and (iii) a manufacturing time of less than 10 weeks from cell isolation to implantation of the graft. We chose ASCs as the source of autologous chondroprogenitor because adipose tissue would be available at the time of the syndactyly release without additional morbidity. While SVF cells and their expanded counterpart (ASCs) are not skeletal cells (SSCs), several studies have demonstrated that these cells can acquire SSCs traits when adequately primed *in vitro*^43^ and form bone organs via ECO when implanted *in vivo.*^20,21,23,24,44^ Moreover, a rapid isolation and expansion process makes ASCs a well-suited cell source for pediatric TE application where donor sites are very limited. In this study, for the three pediatric donors, we obtained on average 2.1 ± 1.1 × 10^6^ SVF cells per gram of adipose tissue (adipose tissues ranging from 2 to 6g), consistent with a recent report on pediatric patients with osteogenesis imperfecta.^45^ After *in vitro* expansion, we generated more than 150 × 10^6^ ASCs within 2 weeks which—using the manufacturing protocol developed here—could potentially generate up to 50 phalangeal grafts.

One limitation of this work is the reduced number of pediatric donors tested in the herein study (*n* *=* *3* for the ECO potential of pediatric ASC and *n* *=* *1* for the phalangeal HCTs grafts). The lack of additional donors is linked to the fact that pediatric biopsies are rare and that we could not obtain additional donors over the course of the study. However, the high reproducibility of ECO using ASCs and SVF-cells associated with collagen sponges demonstrated in a recent report from our group by Chaaban et al.^24^ and the number of *in vivo* replicates used in the present study mitigate the impact of the single pediatric donor used for validation of the manufacturing protocol.

For bone regeneration, TE strategies following an intramembranous ossification (IMO) pathway—usually comprised of a biomaterial mimicking the bone ECM associated with progenitor cells (e.g., BM-MSCs, SVF cells, ASCs)—are the only ones currently evaluated in clinical trials. While promising, this approach has not yielded the expected results. A major limitation for their success remains the lack of vascularization of such large grafts leading to the development of a necrotic core after implantation.^46,47^ Therefore, targeting bone regeneration via the ECO pathway appears to be a more promising approach to generate up-scaled grafts. In fact, hypertrophic chondrocytes are not only more suited for the post-implantation avascular environments but also actively promoting angiogenesis. In addition, transplanted hypertrophic chondrocytes can be replaced by host osteoblasts when they undergo apoptosis or necrosis, mimicking the developmental process.^10,46,47^ Nevertheless, despite reports highlighting the reproducibility and efficacy of ECO-based strategies in preclinical animal models (for review^47,48^), they have not yet reached the stage of clinical trials. This is mostly because the upscaling required to produce grafts of clinically-relevant size remains a major hurdle for the translation of such ECO strategies, where chondroprogenitors first condense and later deposit ECM which gets enriched in GAG over time.^49^ Especially during the condensation phase, maintaining a stable, desired shape and size for a TE graft is challenging. Several approaches are being explored to tackle this issue. A modular strategy consisting in aggregating cartilage microtissues to achieve either larger or more complex tissue architecture, requiring a large number of cells. For example, Hall et al.^50^ report the fabrication of osteochondral grafts by combining iPSC-derived cartilage microtissues in combination with pre-hypertrophic cartilage organoids derived from PDCs. Whereas, Burdis et al.^51^ describe the biofabrication of osteochondral constructs obtained by spatially localizing phenotypically distinct cartilage microtissues within an instructive 3D printed polymer framework. In this case the authors estimated that a human clinical situation would require approximately 330 × 10^6^ BMSCs, 112 × 10^6^ Fat-pad stromal cells and 37 × 10^6^ chondrocytes.

In this study, we chose to modulate the size and architecture of collagen type I sponges in order to achieve HCT-grafts resembling small phalangeal bones (in size and shape). First, we selected the Zimmer plug^®^ because of the similarity in composition with the Ultrafoam^®^ collagen sponge used in previous studies^20,24,42^ and a 40-fold increase in volume. Second, we chose the Optimaix 3D^®^, which owing to its highly oriented fibers was capable to retain its shape and size during the *in vitro* phase. When associated with chondroprogenitors, both of these large collagen sponges supported chondrogenesis with an earlier cartilage onset in the Optimaix 3D^®^ group (Figure 3). However, one major limitation of those Optimaix-based constructs was the lack of remodeling of the collagen sponge *in vitro* and *in vivo* that appeared much less permissive to host cells upon implantation and thus strongly reduced the amount of bone formation (Figure 4). This result validated the Zimmer plug^®^ as the suitable scaffolding material for our proof-of-concept with pediatric ASCs. When used in combination with adult SVF cells, we generated clinically relevant phalangeal grafts (up to 390 mm^3^) containing large cartilage pockets, comparable in size to the one reported by Sheehy et al.,^29^ engineered by using distal phalanx-molded BM-MSC-laden alginate hydrogels, primed toward hypertrophic cartilage and supplemented with a layer of engineered hyaline cartilage. One obvious limitation of this work is the reduced number of pediatric donors—linked to extreme scarcity of pediatric biopsies—tested in the herein study (n = 4 for the ECO potential of pediatric ASC and n = 1 for the phalangeal HCTs grafts). However, the number of *in vivo* replicates *(N* *=* *8)* used for the phalangeal HCT grafts aimed at mitigating the impact of the single pediatric donor used for validation. A relevant drawback of our upscaling method was the inferior cartilage maturation obtained as compared to the one obtained with Ultrafoam^®^ scaffolds of smaller sizes. Nonetheless, when implanted for 12 weeks in an ectopic mouse model mimicking the clinical scenario of an empty phalangeal pocket, such large HCTs were able to *de novo* generate a full bone with preserved shape and size. An interesting aspect of this study was the extent of bone remodeling obtained in these large HCTs, that surpasses the one described in previous reports using ASCs^20,21,23^ or BM-MSCs.^29^ Not only significant amounts of bone and BM tissues were observed, but also large pockets of vascularized cartilage. At that stage also, the collagen scaffolding material was mostly remodeled. Therefore, it is reasonable to speculate that the endochondral ossification was still ongoing rather than yet fully completed.

## Conclusion

In the present study, we generated the proof of concept of an autologous TE strategy to generate osteogenic phalangeal grafts following a developmentally inspired approach for the treatment of children suffering from symbrachydactyly. Specifically, we could show the ECO potential of pediatric ASCs capable of generating *de novo* bone organs of size and shape suited for the targeted clinical scenario. In this context, the *in vitro* generated HCTs phalangeal grafts—owing to their biomechanical properties—would restore the hand pinching capability shortly after the surgery. However, the reduced chondrogenicity associated with the necessary expansion of ASCs should prompt us to better refine the *in vitro* manufacturing protocol in order to achieve better cartilage formation and bone formation. For example, the use of optimized scaffolding materials promoting cell distribution, cartilage formation and bone remodeling would enhance ECO processes. Finally, in the field of pediatric bone repair further research is needed in order to generate TE grafts that would not only (re)generate bone but also support the child development by growing in concert.

## Supplemental Material

sj-docx-1-tej-10.1177_20417314241257352 – Supplemental material for Engineered phalangeal grafts for children with symbrachydactyly: A proof of concept Supplemental material, sj-docx-1-tej-10.1177_20417314241257352 for Engineered phalangeal grafts for children with symbrachydactyly: A proof of concept by Romain Schaller, Adrien Moya, Gangyu Zhang, Mansoor Chaaban, Robert Paillaud, Ewelina M Bartoszek, Dirk J Schaefer, Ivan Martin, Alexandre Kaempfen and Arnaud Scherberich in Journal of Tissue Engineering

sj-png-2-tej-10.1177_20417314241257352 – Supplemental material for Engineered phalangeal grafts for children with symbrachydactyly: A proof of concept Supplemental material, sj-png-2-tej-10.1177_20417314241257352 for Engineered phalangeal grafts for children with symbrachydactyly: A proof of concept by Romain Schaller, Adrien Moya, Gangyu Zhang, Mansoor Chaaban, Robert Paillaud, Ewelina M Bartoszek, Dirk J Schaefer, Ivan Martin, Alexandre Kaempfen and Arnaud Scherberich in Journal of Tissue Engineering

sj-png-3-tej-10.1177_20417314241257352 – Supplemental material for Engineered phalangeal grafts for children with symbrachydactyly: A proof of concept Supplemental material, sj-png-3-tej-10.1177_20417314241257352 for Engineered phalangeal grafts for children with symbrachydactyly: A proof of concept by Romain Schaller, Adrien Moya, Gangyu Zhang, Mansoor Chaaban, Robert Paillaud, Ewelina M Bartoszek, Dirk J Schaefer, Ivan Martin, Alexandre Kaempfen and Arnaud Scherberich in Journal of Tissue Engineering

sj-png-4-tej-10.1177_20417314241257352 – Supplemental material for Engineered phalangeal grafts for children with symbrachydactyly: A proof of concept Supplemental material, sj-png-4-tej-10.1177_20417314241257352 for Engineered phalangeal grafts for children with symbrachydactyly: A proof of concept by Romain Schaller, Adrien Moya, Gangyu Zhang, Mansoor Chaaban, Robert Paillaud, Ewelina M Bartoszek, Dirk J Schaefer, Ivan Martin, Alexandre Kaempfen and Arnaud Scherberich in Journal of Tissue Engineering

## References

[1] Woodside JC Light TR. Symbrachydactyly – diagnosis, function, and treatment. J Hand Surg Am 2016; 41: 135–43; quiz 143. 26254946 10.1016/j.jhsa.2015.06.114

[2] Goodell PB Bauer AS Sierra FJA , et al. Symbrachydactyly. Hand 2016; 11: 262–270. 27698626 10.1177/1558944715614857 PMC5030846

[3] DiGiovanni CW Lin SS Baumhauer JF , et al. Recombinant human platelet-derived growth factor-BB and beta-tricalcium phosphate (rhPDGF-BB/β-TCP): an alternative to autogenous bone graft. J Bone Joint Surg Am 2013; 95: 1184–1192. 23824386 10.2106/JBJS.K.01422

[4] Bartsch A Nikkhah D Miller R , et al. Correction of symbrachydactyly: a systematic review of surgical options. Syst Rev 2023; 12: 218. 37974291 10.1186/s13643-023-02362-7 PMC10652478

[5] Buck-Gramcko D. Symbrachydactyly: a clinical entity. Tech Hand Up Extrem Surg 1999; 3: 242–258. 16609419

[6] Garagnani L Gibson M Smith PJ , et al. Long-term donor site morbidity after free nonvascularized toe phalangeal transfer. J Hand Surg Am 2012; 45(2): 154.e1–154.e7. 10.1016/j.jhsa.2011.12.010 22305432

[7] Patterson RW Seitz WH. Nonvascularized toe phalangeal transfer and distraction lengthening for symbrachydactyly. J Hand Surg Am 2010; 35: 652–658. 20353864 10.1016/j.jhsa.2010.01.011

[8] Sabapathy SR Mohan M Shanmugakrishnan RR. Nonvascularized free toe phalangeal transfers in congenital hand differences: radiological, functional, and patient/parent-reported outcomes. J Hand Surg Am 2021; 46: 1124.e1–1124.e9. 10.1016/j.jhsa.2021.03.012 33966936

[9] Lenas P Moos M Luyten FP. Developmental engineering: a new paradigm for the design and manufacturing of cell-based products. Part I: from three-dimensional cell growth to biomimetics of in vivo development. Tissue Eng Part B Rev 2009; 15: 381–394. 19505199 10.1089/ten.TEB.2008.0575

[10] Mackie EJ Ahmed YA Tatarczuch L , et al. Endochondral ossification: how cartilage is converted into bone in the developing skeleton. Int J Biochem Cell Biol 2008; 40(1): 46–62. 17659995 10.1016/j.biocel.2007.06.009

[11] Chan CKF Chen CC Luppen CA , et al. Endochondral ossification is required for haematopoietic stem-cell niche formation. Nature 2009; 457: 490–494. 19078959 10.1038/nature07547 PMC2648141

[12] Kronenberg HM. Developmental regulation of the growth plate. Nature 2003; 423: 332–336. 12748651 10.1038/nature01657

[13] Deschaseaux F Sensébé L Heymann D. Mechanisms of bone repair and regeneration. Trends Mol Med 2009; 15: 417–429. 19740701 10.1016/j.molmed.2009.07.002

[14] Jukes JM Both SK Leusink A , et al. Endochondral bone tissue engineering using embryonic stem cells. Proc Natl Acad Sci USA 2008; 105: 6840–6845. 18467492 10.1073/pnas.0711662105 PMC2374550

[15] Kuhn LT Liu Y Boyd NL , et al. Developmental-like bone regeneration by human embryonic stem cell-derived mesenchymal cells. Tissue Eng Part A 2014; 20: 365–377. 23952622 10.1089/ten.tea.2013.0321 PMC4350005

[16] Scotti C Tonnarelli B Papadimitropoulos A , et al. Recapitulation of endochondral bone formation using human adult mesenchymal stem cells as a paradigm for developmental engineering. Proc Natl Acad Sci USA 2010; 107(16): 7251–7256. 20406908 10.1073/pnas.1000302107 PMC2867676

[17] McDermott AM Herberg S Mason DE , et al. Recapitulating bone development through engineered mesenchymal condensations and mechanical cues for tissue regeneration. Sci Transl Med 2019; 11: 1–16. 10.1126/scitranslmed.aav7756 PMC6959418 31167930

[18] Bolander J Ji W Leijten J , et al. Healing of a large long-bone defect through serum-free in vitro priming of human periosteum-derived cells. Stem Cell Rep 2017; 8: 758–772. 10.1016/j.stemcr.2017.01.005 PMC5355567 28196691

[19] Nilsson Hall G Mendes LF Gklava C , et al. Developmentally engineered callus organoid bioassemblies exhibit predictive in vivo long bone healing. Adv Sci 2020; 7: 1–16. 10.1002/advs.201902295 PMC6974953 31993293

[20] Osinga R Di Maggio N Todorov A , et al. Generation of a bone organ by human adipose-derived stromal cells through endochondral ossification. Stem Cells Transl Med 2016; 5(8): 1090–1097. 27334490 10.5966/sctm.2015-0256 PMC4954448

[21] Guerrero J Pigeot S Müller J , et al. Fractionated human adipose tissue as a native biomaterial for the generation of a bone organ by endochondral ossification. Acta Biomater 2018; 77: 142–154. 30126590 10.1016/j.actbio.2018.07.004

[22] Weiss-Bilka HE McGann ME Meagher MJ , et al. Ectopic models for endochondral ossification: comparing pellet and alginate bead culture methods. J Tissue Eng Regen Med 2018; 12: e541–e549. 10.1002/term.2324 PMC5773408 27690279

[23] Huang R-L Guerrero J Senn AS , et al. Dispersion of ceramic granules within human fractionated adipose tissue to enhance endochondral bone formation. Acta Biomater 2020; 102: 458–467. 31783141 10.1016/j.actbio.2019.11.046

[24] Chaaban M Moya A García-García A , et al. Harnessing human adipose-derived stromal cell chondrogenesis in vitro for enhanced endochondral ossification. Biomaterials 2023; 303: 122387. 37977007 10.1016/j.biomaterials.2023.122387

[25] Oliveira SM Mijares DQ Turner G , et al. Engineering endochondral bone: in vivo studies. Tissue Eng Part A 2009; 15: 635–43. 10.1089/ten.tea.2008.0052 PMC2751848 18759673

[26] Robla Costales D Junquera L García Pérez E , et al. Ectopic bone formation during tissue-engineered cartilage repair using autologous chondrocytes and novel plasma-derived albumin scaffolds. J Craniomaxillofac Surg 2016; 44: 1743–1749. 27618716 10.1016/j.jcms.2016.08.005

[27] Pigeot S Klein T Gullotta F , et al. Manufacturing of human tissues as off-the-shelf grafts programmed to induce regeneration. Adv Mater 2021; 33: e2103737. 10.1002/adma.202103737 PMC11468960 34486186

[28] Pigeot S Bourgine PE Claude J , et al. Orthotopic bone formation by streamlined engineering and devitalization of human hypertrophic cartilage. Int J Mol Sci 2020; 21: 1–14. 10.3390/ijms21197233 PMC7582540 33008121

[29] Sheehy EJ Mesallati T Kelly L , et al. Tissue engineering whole bones through endochondral ossification: regenerating the distal phalanx. Biores Open Access 2015; 4: 229–241. 26309799 10.1089/biores.2015.0014 PMC4540120

[30] Keane TJ Badylak SF. Biomaterials for tissue engineering applications. Semin Pediatr Surg 2014; 23: 112–118. 24994524 10.1053/j.sempedsurg.2014.06.010

[31] Mumme M Wixmerten A Miot S , et al. Tissue engineering for paediatric patients. Swiss Med Wkly 2019; 149: w20032. 30950502 10.4414/smw.2019.20032

[32] Mareschi K Ferrero I Rustichelli D , et al. Expansion of mesenchymal stem cells isolated from pediatric and adult donor bone marrow. J Cell Biochem 2006; 97: 744–754. 16229018 10.1002/jcb.20681

[33] Knuth CA Kiernan CH Palomares Cabeza V , et al. Isolating pediatric mesenchymal stem cells with enhanced expansion and differentiation capabilities. Tissue Eng Part C Methods 2018; 24: 313–321. 29631483 10.1089/ten.TEC.2018.0031

[34] D’Ippolito G Schiller PC Ricordi C , et al. Age-related osteogenic potential of mesenchymal stromal stem cells from human vertebral bone marrow. J Bone Miner Res 1999; 14: 1115–1122. 10404011 10.1359/jbmr.1999.14.7.1115

[35] Marolt Presen D Traweger A Gimona M , et al. Mesenchymal stromal cell-based bone regeneration therapies: from cell transplantation and tissue engineering to therapeutic secretomes and extracellular vesicles. Front Bioeng Biotechnol 2019; 7: 352. 31828066 10.3389/fbioe.2019.00352 PMC6890555

[36] Feisst V Meidinger S Locke MB. From bench to bedside: use of human adipose-derived stem cells. Stem Cells Cloning 2015; 8: 149–162. 26586955 10.2147/SCCAA.S64373 PMC4636091

[37] Argentati C Morena F Bazzucchi M , et al. Adipose stem cell translational applications: from bench-to-bedside. Int J Mol Sci 2018; 19: 2475. 10.3390/ijms19113475 PMC6275042 30400641

[38] Cheng C Chaaban M Born G , et al. Repair of a rat mandibular bone defect by hypertrophic cartilage grafts engineered from human fractionated adipose tissue. Front Bioeng Biotechnol 2022; 10: 841690. 35350180 10.3389/fbioe.2022.841690 PMC8957819

[39] Güven S Mehrkens A Saxer F , et al. Engineering of large osteogenic grafts with rapid engraftment capacity using mesenchymal and endothelial progenitors from human adipose tissue. Biomaterials 2011; 32: 5801–5809. 21605897 10.1016/j.biomaterials.2011.04.064

[40] Power L Acevedo L Yamashita R , et al. Deep learning enables the automation of grading histological tissue engineered cartilage images for quality control standardization. Osteoarthr Cartil 2021; 29: 433–443. 10.1016/j.joca.2020.12.018 33422705

[41] Bankhead P Loughrey MB Fernández JA , et al. QuPath: open source software for digital pathology image analysis. Sci Rep 2017; 7: 16878. 29203879 10.1038/s41598-017-17204-5 PMC5715110

[42] Scotti C Piccinini E Takizawa H , et al. Engineering of a functional bone organ through endochondral ossification. Proc Natl Acad Sci USA 2013; 110(10): 3997–4002. 23401508 10.1073/pnas.1220108110 PMC3593845

[43] Hennig T Lorenz H Thiel A , et al. Reduced chondrogenic potential of adipose tissue derived stromal cells correlates with an altered TGFbeta receptor and BMP profile and is overcome by BMP-6. J Cell Physiol 2007; 211: 682–691. 17238135 10.1002/jcp.20977

[44] Janicki P Kasten P Kleinschmidt K , et al. Chondrogenic pre-induction of human mesenchymal stem cells on β-TCP: enhanced bone quality by endochondral heterotopic bone formation. Acta Biomater 2010; 6: 3292–3301. 20123138 10.1016/j.actbio.2010.01.037

[45] Tauer JT Al-Jallad H Umebayashi M , et al. Characterization and functional analysis of the adipose tissue-derived stromal vascular fraction of pediatric patients with osteogenesis imperfecta. Sci Rep 2022; 12: 2414. 10.1038/s41598-022-06063-4 PMC8844034 35165317

[46] Thompson EM Matsiko A Farrell E , et al. Recapitulating endochondral ossification: a promising route to in vivo bone regeneration. J Tissue Eng Regen Med 2015; 9: 889–902. 24916192 10.1002/term.1918

[47] Nadine S Fernandes IJ Correia CR , et al. Close-to-native bone repair via tissue-engineered endochondral ossification approaches. iScience 2022; 25: 105370. 36339269 10.1016/j.isci.2022.105370 PMC9626746

[48] Sheehy EJ Kelly DJ O’Brien FJ. Biomaterial-based endochondral bone regeneration: a shift from traditional tissue engineering paradigms to developmentally inspired strategies. Mater today Bio 2019; 3: 100009. 10.1016/j.mtbio.2019.100009 PMC7061547 32159148

[49] Goldring MB Tsuchimochi K Ijiri K. The control of chondrogenesis. J Cell Biochem 2006; 97: 33–44. 16215986 10.1002/jcb.20652

[50] Hall GN Tam WL Andrikopoulos KS , et al. Patterned, organoid-based cartilaginous implants exhibit zone specific functionality forming osteochondral-like tissues in vivo. Biomaterials 2021; 273: 120820. 33872857 10.1016/j.biomaterials.2021.120820

[51] Burdis R Chariyev-Prinz F Browe DC , et al. Spatial patterning of phenotypically distinct microtissues to engineer osteochondral grafts for biological joint resurfacing. Biomaterials 2022; 289: 121750. 36084483 10.1016/j.biomaterials.2022.121750

