# Supplementary material for: Engineered phalangeal grafts for children with symbrachydactyly: A proof of concept
Source: J Tissue Eng. 2024 Jun 12;15:20417314241257352. doi: 10.1177/20417314241257352 (PMC11171439; doi:10.1177/20417314241257352)
Supplement: sj-docx-1-tej-10.1177_20417314241257352 – Supplemental material for Engineered phalangeal grafts for children with symbrachydactyly: A proof of concept [file sj-docx-1-tej-10.1177_20417314241257352.docx]

**FIGURES LEGENDS**

**Figure 1. Pediatric ASCs recapitulate endochondral ossification *in vivo* when adequately primed *in vitro.***

**(A)** Schematic overview of the experimental design. **(B)** 0.5x10^6^ Pediatric ASC P1 cells are associated with a collagen scaffold (cylindrical shape, 4mm diameter and 3mm height; *V*= 37.68mm^3^) and cultured *in vitro* for 4W (3 weeks chondrogenic + 1 week hypertrophy). Representative images of **(a-d)** Safranin-O staining, cartilage tissue in red, mineralized cartilage in blue and non-cartilage tissue in green, **(e-h)** collagen type II and **(i-l)** collagen type X of the *in vitro* generated cartilage tissues. **(C)** *In vivo* bone remodeling via endochondral ossification after 12 weeks of ectopic implantation in a nude mice model. Representative images of **(a-d)** Microcomputed tomography (µCT), Bone (dense signal with smooth surface) mineralized HCT (signal with a rough surface). **(e-h)** Safranin-O staining, bone (dark green), fibrotic tissue (light green), cartilage (red), bone marrow (purple). *(N = 13, 2-4 biological replicates per donor, 4 donors tested).* Black scale bar = 500µm, white scale bar = 1mm, red scale bar 100µm. Symbols: b: Bone, BM: Bone Marrow, c: cartilage, s: scaffold, *: blood vessel.

**Figure 2. Adult SVF cells associated large collagen sponge scaffolds generate phalangeal HCT grafts *in vitro*.**

**(A**) Schematic overview of the experimental design and macroscopic views of the phalangeal HCT grafts **(a) (i)** before and **(ii)** after cell seeding and **(b)** throughout the *in vitro* cartilage maturation. Histological assessments of **(B)** 0.9x10^6^ SVF cells (6000 cell/mm^3^) associated with Optimaix® collagen scaffold (bar shape, 10 mm length 5 mm width and 3 mm height; *V*= 150 mm^3^) or **(C)** 3x10^6^ SVF cells (2000 cell/mm^3^) associated with Zimmer Plug® collagen scaffold (cylindrical shape, 10 mm diameter and 20 mm length; *V*= 1500 mm^3^) and cultured *in vitro* for **(a-c)** 4W (3 weeks chondrogenic + 1 week hypertrophy), **(d-f)** 5W (4 weeks chondrogenic + 1 week hypertrophy) or **(g-i)** 7W (6 weeks chondrogenic + 1 week hypertrophy). Representative images of **(a,d,g)** Safranin-O staining, **(b,e,h)** collagen type II and **(c,f,h)** collagen type X obtained from the *in vitro* generated HCT grafts. *(N ≥ 1, 1-2 biological replicates per donor, 1-2 donors tested).* Red scale bar = 10mm, black scale bar = 1mm, yellow scale bar = 150µm.

**Figure 3. *In vitro* evolution of the size and cartilage maturation of phalangeal HCTs grafts generated from adult SVF cells.**

**(A)** Time course of the estimated volumes of the phalangeal HCT grafts for both the Zimmer Plug® and Optimaix® groups. Data are expressed as mean ± SD (mm^3^). *(N = 6, 6 biological replicates per donor, 2 donors tested per scaffolding material).* **(B)** Automated histological assessment of the *in vitro* cartilage maturation for the 4W-, 5W- and 7W-groups. Safranin-O-stained pictures are cut into square tiles (336 pixels by 336 pixels) and scored 0-non cartilaginous tissue; (2) poor cartilage maturation; (4) moderate cartilage maturation; (6) high cartilage maturation. Distribution of the number of tiles for each cartilage maturation category expressed as a percentage of the total number of tiles and plotted as a histogram. *(N ≥ 1, 1-2 biological replicates per donor, 1-2 donors tested).*

**Figure 4. Phalangeal HCT grafts remodel into bone organs via endochondral ossification *in vivo*.**

Bone remodeling phenotypes observed for **(A)** 7W-Optimaix- and **(B)** 7W-Zimmer-based grafts after 12 weeks of implantation *in vivo*. **(a)** Macroscopic and Micro- µCT views. Representative images of **(b)** Safranin-O and **(c)** H&E sections. **(d)** Automated tissue segmentation performed H&E sections using a semi-automated script on QuPath. **(C)** Evolution of mineralized tissue (mm^3^) obtained by µCT after 12 weeks *in vivo*. Data are expressed as mean ± SD *(N ≥ 4, 4 biological replicates per donor, 1-2 donors tested)*. Evolution of **(D)** Bone and Bone Marrow, **(E)** Cartilage and **(F)** Other tissues within the H&E sections obtained after 12 weeks *in vivo*. Data are expressed as a percentage of the total tissue mean ± SD. *(N ≥, 4 biological replicates per donor, 1-2 donors tested).* b: Bone, BM: Bone Marrow, c: cartilage, s: scaffold, *: blood vessel. Red scale bar = 10 mm, black scale bar = 1mm, yellow scale bar = 150µm. ********p<0.05 Mann Whitney test (Zimmer group vs. Optimaix group);* ***^##^*** *p<0.01 Mann Whitney test (7 weeks vs. 5 weeks).*

**Figure 5. Envisioned manufacturing strategy for autologous phalangeal HCT grafts production to treat children suffering from Symbrachydactyly.**

**(A**) Schematic overview of the manufacturing protocol. Pediatric SVF cells are expanded for 2 weeks (ASC P1). 3x10^6^ pediatric ASCs P1 are associated with Zimmer Plug® collagen scaffolds and cartilage maturation is induced for 7 weeks. **(B)** Macroscopic view of the cartilage constructs after **(a)** 1 week and **(b)** 7 weeks of cartilage maturation. **(C)** Representative images of Safranin-O staining, **(D) (a)** collagen type II and **(b)** collagen type X obtained from the *in vitro* generated cartilage. **(E)** Time course of the estimated volumes of the HCTs. Data are expressed as mean ± SD (mm^3^). *(N ≥ 4, 4-6 biological replicates per donor, 1-2 donors tested per cell group).* **(F)** Automated histological assessment of the *in vitro* maturation of the cartilage tissues after 7 weeks of induction. *(N ≥ 3, 1-3 biological replicates per donor, 1-2 donors tested).* Red scale bar = 10mm, black scale bar = 1mm, yellow scale bar = 150µm.

**Figure 6. Pediatric phalangeal HCT grafts remodel into bone tissue via endochondral ossification.**

7W Pediatric ASCs P1 based Zimmer constructs were implanted in an ectopic nude mice model for 12 weeks. **(A) (a)** Macroscopic and **(b,c)** Micro- µCT views post-implantation. Representative images of **(B)** H&E and **(C)** Safranin-O staining showing evidence of ECO in the implanted constructs **(a)** whole section, **(b)** early cartilage remodeling with the presence of multinucleated cells, **(c)** Chondrocyte to osteoblast transition, **(d)** Outer cortical bone, **(e)** intermediate cartilage remodeling with the presence of bone and blood vessels and **(f)** mature bone located at the core of the constructs. **(D)** Bone Sialoprotein (BSP) staining **(a)** whole section, **(b)** early cartilage remodeling. **(E)** Mouse and human cells contribution to the ECO. Representative images of human nuclei and Dapi staining of **(a)** large section, **(b)** early cartilage remodeling with the presence of murine multinucleated cells, **(c)** Chondrocyte to osteoblast transition with the presence of human chondrocytes and osteoblasts and **(d)** Outer cortical bone with the presence of murine osteoblasts. Mouse nuclei in blue, and human nuclei in pink on the merged pictures. **(F)** Evolution of mineralized tissue in mm^3^ for pediatric phalangeal HCT grafts obtained by µCT after 12 weeks *in vivo*. Data are expressed as mean ± SD *(N =8, 1 donor tested)*. Evolution of **(G)** Bone and Bone Marrow, **(H)** Other and **(I)** Cartilage obtained by automated tissue segmentation performed H&E sections using a semi-automated script on QuPath. Data are expressed as a percentage of the total tissue mean ± SD. *(N = 6, 1 donor tested).* Symbols: b: bone, c: cartilage, s: scaffold, *: blood vessel, yellow circles murine multinucleated cells, white arrows human osteoblasts. White scale bar = 1mm, black scale bar = 1mm, blue scale bar = 500µm, yellow scale bar = 150µm, green scale bar = 20µm.

**Supplementary Figure 1. Higher cell seeding density impairs SVF cells chondrogenic capacity when associated with Zimmer Plug® collagen *in vitro*.**

**(A)** Histological assessments of 0.9x10^6^ SVF cells (6000 cell/mm^3^) associated with Zimmer Plug® collagen scaffold (cylindrical shape, 10 mm diameter and 20 mm length; *V*= 1500 mm^3^) and cultured *in vitro* for 7 weeks. Representative images of **(a)** Safranin-O staining and **(b)** collagen type II. **(B)** Macroscopic view of the cartilage constructs after **(a)** 1 week and **(b)** 7 weeks of cartilage maturation **(C)** Time course of the estimated volumes of the HCTs. Data are expressed as mean ± SD (mm^3^). *(N ≥ 4, 4-6 biological replicates per conditions, 1 donor tested).* Red scale bar = 10mm, black scale bar = 1mm, yellow scale bar = 150µm.

**Supplementary Figure 2. Endochondral ossification of 5W-Optimaix® and -Zimmer Plug® phalangeal HCT grafts *in vivo*.**

Partial bone remodeling phenotypes observed for **(A)** 5W-Optimaix-based constructs and **(B)** 5W-Zimmer based constructs after 12 weeks of implantation *in vivo*. **(a)** Macroscopic and Micro- µCT views. Representative images of **(b)** Safranin-O and **(c)** H&E sections. **(d)** Automated tissue segmentation performed H&E sections using a semi-automated script on QuPath. *(N = 4, 4 biological replicates, 1 donor tested).* Symbols b: bone, c: cartilage, s: scaffold, *: blood vessel. Red scale bar = 10mm, white scale bar = 1mm, black scale bar = 1mm, yellow scale bar = 150µm.

**Supplementary Figure 3. Implanted HCTs from pediatric donor produce bone via endochondral ossification while preserving cartilage tissue *in vivo*.**

**(A-C)** Representative images of (a-b) Safranin-O stained and (c-d) BSP stained of 3 out of 6 replicates of 7W pediatric phalangeal HCT grafts after 12 weeks of *in vivo* implantation. **(a, c)** Whole section, **(b, d)** Cartilage tissues*.* Black scale bar = 1mm, yellow scale bar = 150µm. Symbols: b: Bone, c: cartilage.
