# Supplementary figures and images for: Engineered phalangeal grafts for children with symbrachydactyly: A proof of concept
Source: J Tissue Eng. 2024 Jun 12;15:20417314241257352. doi: 10.1177/20417314241257352 (PMC11171439; doi:10.1177/20417314241257352)

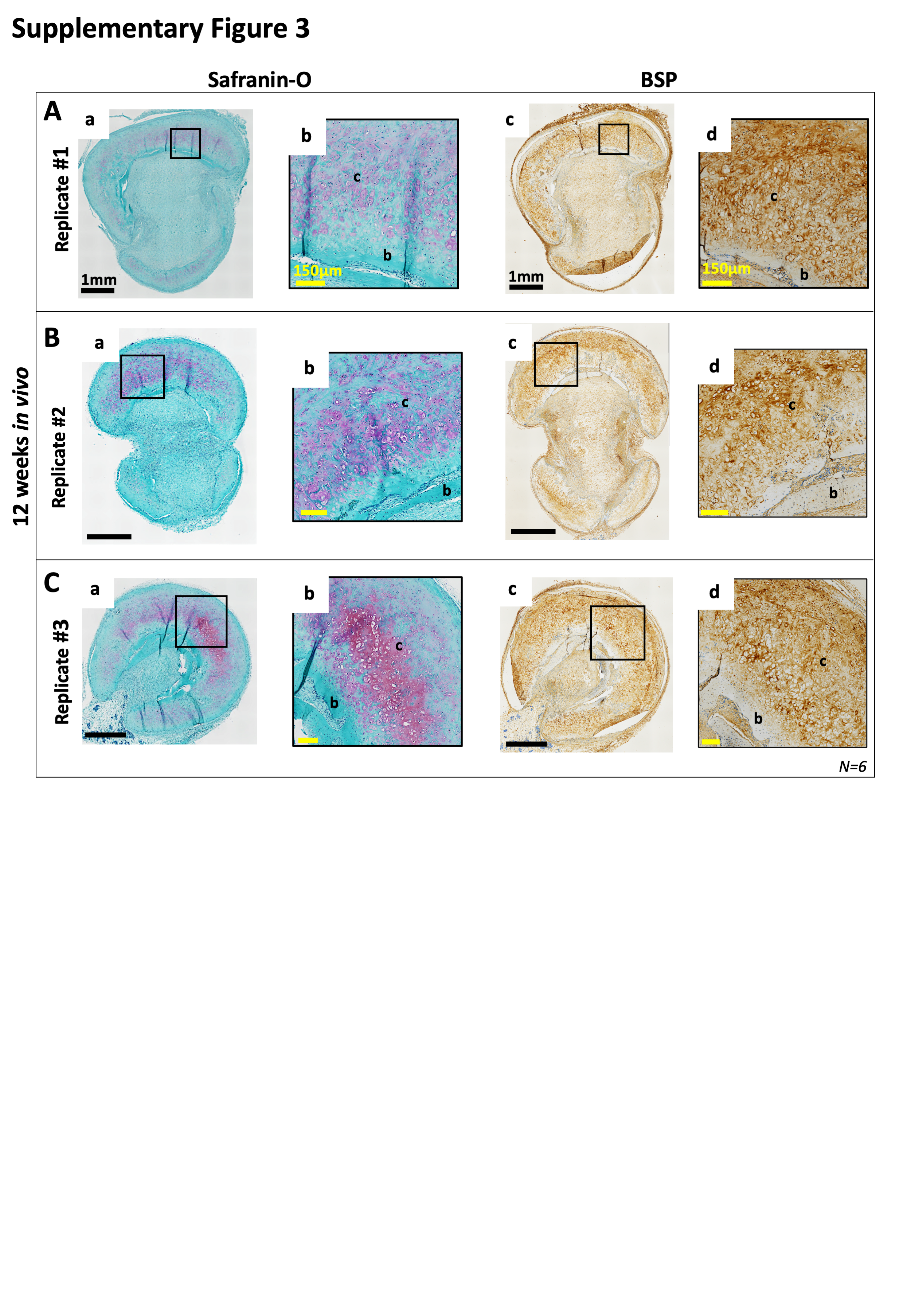

Supplement: sj-png-2-tej-10.1177_20417314241257352 – Supplemental material for Engineered phalangeal grafts for children with symbrachydactyly: A proof of concept [file sj-png-2-tej-10.1177_20417314241257352.png]

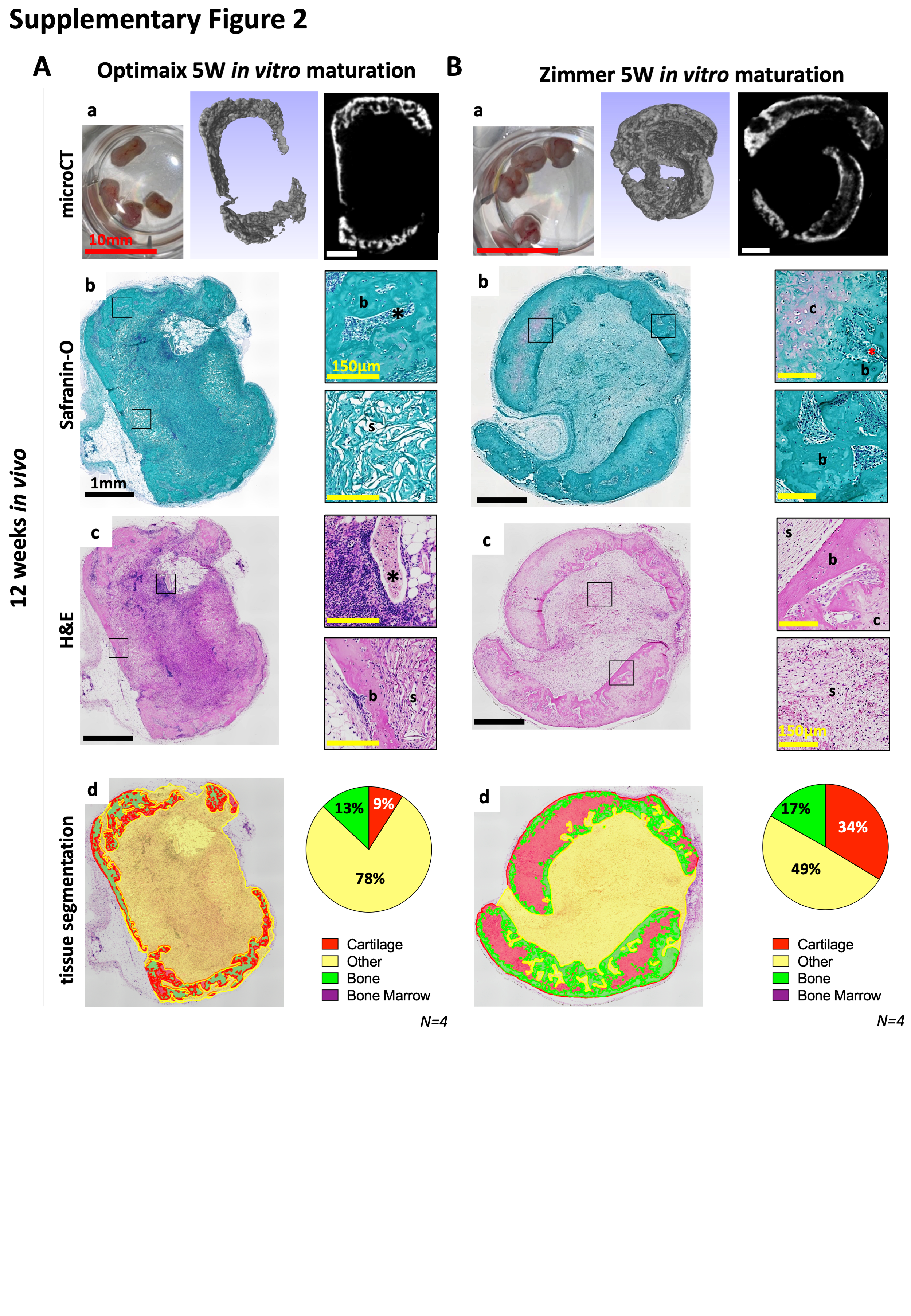

Supplement: sj-png-3-tej-10.1177_20417314241257352 – Supplemental material for Engineered phalangeal grafts for children with symbrachydactyly: A proof of concept [file sj-png-3-tej-10.1177_20417314241257352.png]

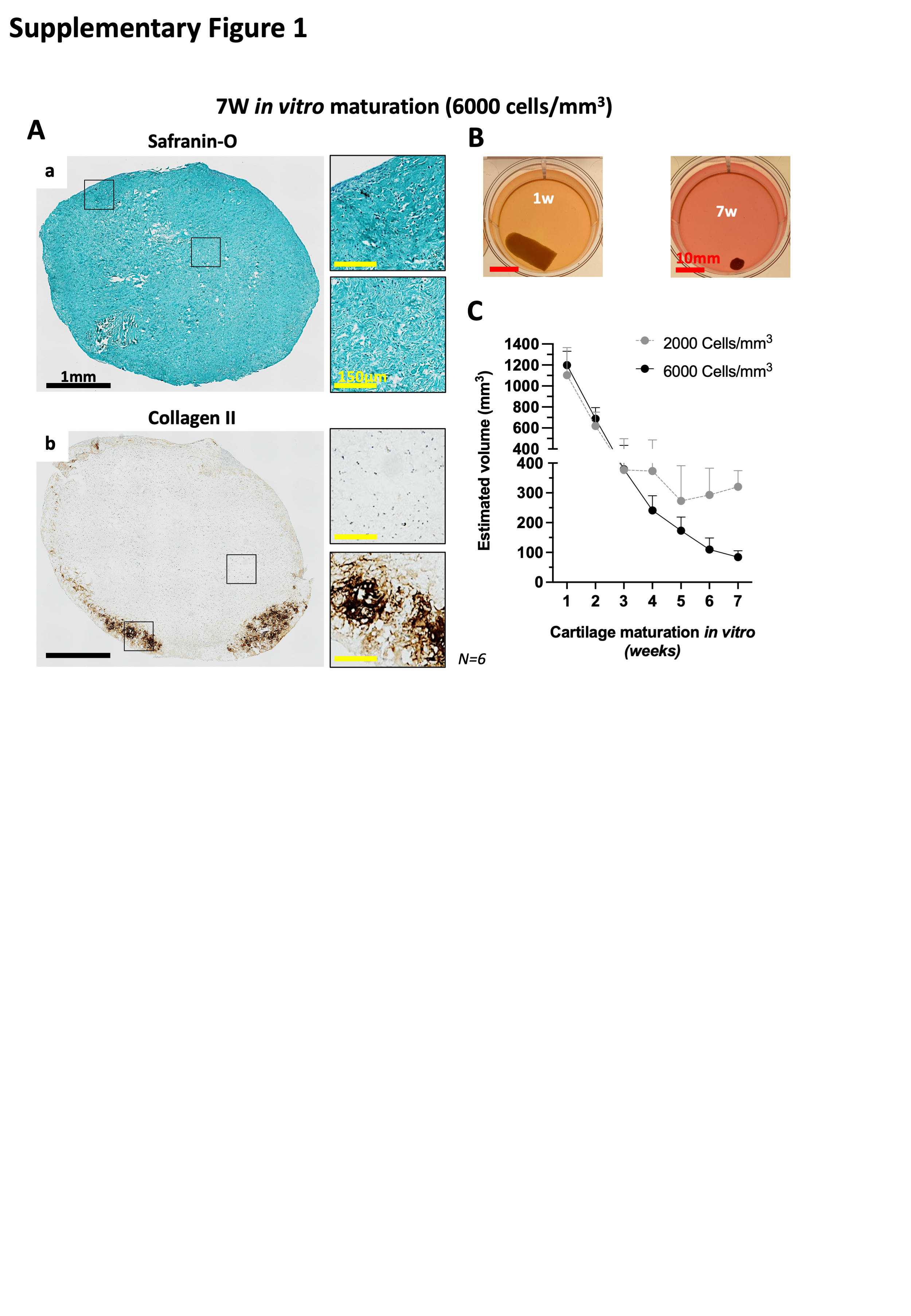

Supplement: sj-png-4-tej-10.1177_20417314241257352 – Supplemental material for Engineered phalangeal grafts for children with symbrachydactyly: A proof of concept [file sj-png-4-tej-10.1177_20417314241257352.png]
